# Task Control Deficit in Individuals With Non-suicidal Self-Injury

**DOI:** 10.3389/fpsyt.2021.608357

**Published:** 2021-02-05

**Authors:** Seo Jeong Lee, Myoung Ho Hyun

**Affiliations:** ^1^Department of Psychiatry, Chung-Ang University Hospital, Seoul, South Korea; ^2^Department of Psychology, Chung-Ang University, Seoul, South Korea

**Keywords:** task control, objective-interference effect, non-suicidal self-injury, executive control, non-verbal Stroop task

## Abstract

**Background:** Numerous people in clinical settings who have experienced repeated self-injuries explain their non-suicidal self-injury (NSSI) as “habitual” or due to “difficulty avoiding impulses related to NSSI.” Previous studies present retrospective reports, where they experience frequent self-injurious urges and try to resist but fail. However, no study has directly investigated repeated behavioral control problems of people who engage in chronic NSSI through behavioral measurements in an experimental setting. The current study sought to investigate whether people who repeatedly attempt NSSI demonstrate deficiency in task control ability called the object-interference (O-I effect).

**Methods:** The current study performed object interference tasks on 90 participants, of which 45 were those who reported repeated NSSI while 45 comprised the control group.

**Results:** We observed delayed reaction times for object stimulus compared to abstract stimulus in the NSSI group, indicative of the object interference effect. This reflects task control deficits and difficulties in NSSI related behavioral control in the repeated NSSI group. When NSSI tools were additionally presented as a target stimulus, longer reaction times and more errors were observed in the NSSI group compared to the control group.

**Discussion:** The current study discusses the clinical implications of the results from diagnostic point of view and provides suggestions for future research for treatment and prevention.

## Introduction

Self-injurious thoughts and behaviors (SITBs) can be divided into two main types depending on whether suicidal intentions are present (suicidal thoughts, suicidal plans, suicidal attempts) or not (suicidal gestures, non-suicidal self-injury thoughts and behaviors) (1). However, NSSI has been discussed as strong longitudinal predictors of future suicidal attempts (2) and are related to suicides (3). Around 70% of adolescents who engage in NSSI report experiences of suicidal attempt with clear suicidal intentions (4, 5). Furthermore, 91% of those who exhibit clinically serious levels of self-injuries also show mild levels of self-injury (6). Regardless of presence of suicidal intentions or seriousness, SITBs are significant problems that can lead to death by suicide. In this context, one can easily predict that those who engage in chronic NSSI are vulnerable to life-threatening situations.

When asked as to why they engage in non-suicidal self-injury (NSSI), many individuals in clinical settings who have experienced repeated NSSI explain their early experiences of it with functional factors, such as “to get rid of a bad feeling” or “to feel alive.” On the other hand, those with chronic NSSI experiences explain recent NSSI as “as always,” “difficulty avoiding impulses (to indulge in NSSI),” or “habitual.” Early NSSI experience is related to weakened inhibitory control abilities during negative emotional situations and emotion regulation difficulties (7–10), while chronic NSSI is suggested to be related to problems with controlling repeated behaviors (11). This is known to be a task control ability called the O-I effect (12).

Only a few of those who have experienced NSSI stop after one or two experiences. In many cases, NSSI is repeated and becomes chronic. Furthermore, as behavior repeats over time, it becomes fixed through the reinforcement paradigm, and leads to life-threatening NSSI due to increased frequency and severity (13, 14). A recent cognitive neuroscientific model of NSSI explains the process of chronic stages of NSSI as follows: as NSSI experiences repeat, pain and shame decrease, and changes in neural circuits allow for becoming psychologically, physiologically, and physically accustomed to self-injurious behavior, leading to chronic stages of mechanical repetition (15).

According to Monsell (16), a task is activated in two ways: a top-down approach, where a task is planned by an objective or an instruction, and a bottom-up approach, where an associated specific task is activated due to perceiving a stimulus. Here, the O-I effect is a deficit in executive control ability that inhibits automatic behaviors triggered by environmental cues. From the cognitive neuroscientific perspective, habitual behaviors are not innate, but rather a response triggered by specific situations or stimuli. Furthermore, habitual behaviors become fixed over time, and once acquired, only a small amount of effort is required to produce them (16, 17).

Humans are fundamentally able to directly perceive the behavioral meaning of the object (tool). Therefore, looking at specific objects can produce latent motor responses, even without intentions of action (18, 19). In this context, a recent study on “motor evoked potentials” showed that simply viewing a specific target object through a screen activates related motor planning and relevant brain regions (20).

Objects also act in various ways on human behavior depending on the attached meaning. If NSSI is primed with low pain intensity, the perceived pain during the actual NSSI is reduced. In addition, semantic priming strongly relates to NSSI implicitly or causes attention bias; construal priming induces positive attitudes regarding NSSI, or causes the occurrence of NSSI thoughts; and behavior and goal priming allows individuals to look for, or become involved in self-injurious behaviors. Recent studies suggest the media, Internet, and peer group as priming factors for SITBs (Self-injurious thought and behaviors) (21).

**Figure 1:**
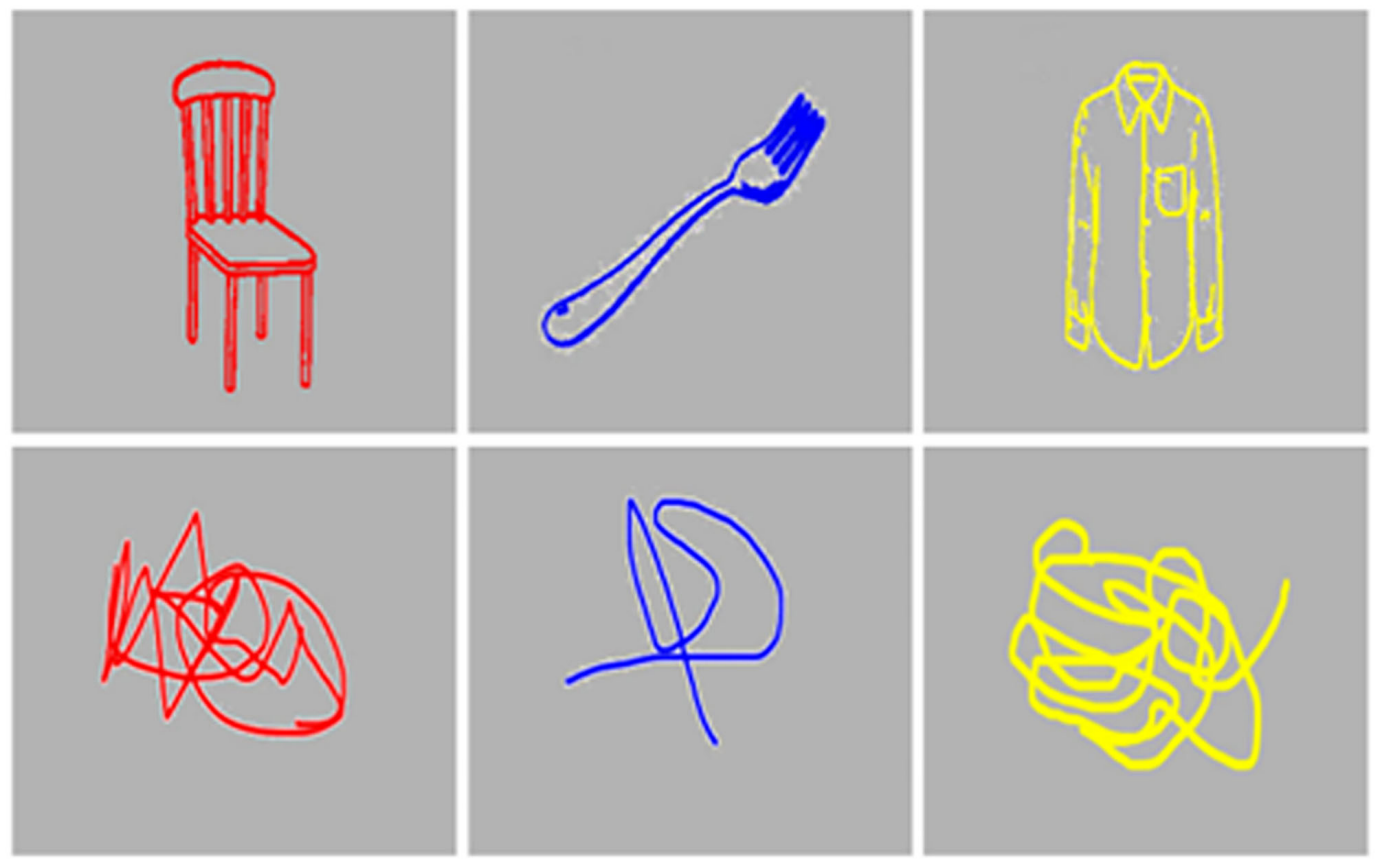
An example of object stimuli and abstract stimuli used in the current study.

According to Prevor and Diamond (12), task control ability refers to a control mechanism that helps resolve task conflict and successfully achieve goal-oriented behavior by appropriately controlling for the task at hand in a situation where multiple tasks are competing. They found the O-I effect during a development process of non-verbal Stroop variant task for children. This task presents a known, named target picture and an abstract picture in colored forms, and requires verbally naming the color of the picture. The task produced interesting results where children took longer to name the color of the meaningful target with a name, compared to naming the color of an abstract target form. For example, in Figure 1, children show slowed response saying “red” when looking at “a red chair” compared to when looking at “a red abstract figure.” This is called the O-I effect (22, 23).

This phenomenon is observed before the age of 6.5 (age 3.5~6.5), and older children or adults can quickly resolve task conflict through maturation of executive control process (22). Children usually exhibit color preference during ages 2–3, and form preferences until the age of 9. Therefore, as age increases, the tendency to recognize the object increases, but the form preference is offset by the increase in the effectiveness of the frontal lobe executive control (23). Therefore, the O-I effect in adults is an abnormal phenomenon, and is strong evidence to indicate low levels of task control.

There are a variety of phenomena of “Stroop-like paradigms,” such as picture-word, or color-word interference effect, etc. The O-I effect is seemingly similar to the original Stroop interference effect, but it is explained by another factor (24). It is not simply a matter of word selection due to lexical interference, but a conflict between task sets of processing color vs. processing object.

La Heij et al. (23) performed several variants of object-interference tasks to identify distinct characteristics of the O-I effect in children. Repeated verification was made in many ways, such as naming the object's color rather than their location, or presenting objects where children recognize their functions but have difficulty naming (e.g., contrabass, monkey-spanner, etc.). The results showed a consistent O-I effect when the color-naming task was changed to a location-naming task, or when the lexical difficulty increased, or even when objects that could not be named were presented. In addition, when required to produce non-verbal responses such as pressing buttons during the original Stroop task, the effects of verbal interference disappear, suggesting a differentiation with simple word selection problems due to verbal interference. In other words, the O-I effect is not simply a semantic priming effect or a word selection problem, but rather the ability to control for the fundamental confusion of task selection between processing either the color or the object, a more comprehensive concept.

The previously mentioned behavioral meaning and motor execution induced by an object have important implications not only for NSSI, but also for mental illness involving repeated behaviors such as obsessive-compulsive behaviors. A recent study on obsessive-compulsive disorder (OCD) patients identified abnormalities in executive control abilities (19). An example is an OCD patient whose main symptom of checking behaviors is repeatedly locking the doorknob. When the patient tries to open the doorknob to go outside, the doorknob triggers the checking behavior of repeatedly locking it. In this situation, the goal-oriented behavior of going outside and the checking behavior triggered by the doorknob cause a “task competition.” In other words, environmental cues trigger behaviors that are repeatedly habituated, which hinder goal-oriented behaviors. This creates difficulties in task control that requires executive control.

Such challenges also apply to repeated NSSI patients. Repeated NSSI patients in clinical settings report that looking at frequently used self-injury tools triggers self-injurious behavior. Furthermore, they state that it is difficult to control NSSI impulses because many objects around them are seen as self-injury tools. In other words, daily goal-oriented activities compete with self-injurious behaviors triggered by objects, thus leading to situations where controlling repeated, habituated self-injurious behaviors is difficult. These fixated NSSI behaviors hinder functional performance in important aspects of life as they are triggered by various target objects met in everyday lives, thus gradually deepening adaptive problems. It is therefore important to remove self-injury tools from those with dangers of self-injury. One study showed that the most effective way of resisting impulses of self-injury is to remove the means of self-injury (tools) used frequently at home (11). However, there is insufficient direct evidence explaining this phenomenon. Hence, repeated NSSI behaviors as an executive control problem of habituated behaviors triggered by objects should be objectively measured. This will allow for the presentation of clear evidence for a treatment protocol to prevent NSSI relapses.

Therefore, the current study aims to see whether participants who report repeated NSSI experience object-induced task conflict to produce the O-I effect, compared to the control group. In Blocks 1 and 2, it can be hypothesized that the NSSI group, compared to the healthy control group, will report longer reaction times to object stimuli than abstract stimuli. It can also be expected that both NSSI group and healthy control group will report relatively higher number of errors to object stimuli than abstract stimuli. Additionally, exploratory attempts were made, where self-injury tools were presented as object stimuli in Block 3, to compare any differences between results from Blocks 1 and 2.

## Methods

### Participants

From March to July 2019, participants were invited from three universities located in Seoul, through both online and offline notifications. The selection criterion was adults who are 18 or older reporting repeated NSSI, and 48 individuals participated. The participants were administered the Self-Injurious Thoughts and Behaviors Interview- Korean (SITBI-K). Those who reported serious NSSI that required medical treatment in the last month were considered high-risk, and were removed as per research ethics. Also, those with formal thought disorder, intellectual disability, and organic mental disorder, etc. who are unable to report reliable self-reports were excluded. Participants in the healthy control group were matched for age and sex with the NSSI group.

### Measures

#### Non-suicidal Module From the SITBI-K

The NSSI module from the SITBI (25) was translated into Korean by the researcher, and was used in the current study (26). Study participants' selection criteria were identified using items for NSSI experience and frequency.

#### Object-Interference Task

The Object-Interference task was computerized by a PhD in electrical engineering using WPF (Window Presentation Foundation) C#, and the program was implemented in Visual Studio 2017 (NET Framework 4.5.2) and was run on a 14-inch laptop. Voice Key (SV-1, cedrus) connected to a headset was used to measure speech production. Verbal responses are suggested to reflect cognitive processing speed more accurately, unlike the motor response (measured with keyboard or keypad) (27, 28).

The Object-Interference task was constructed using the Experiment 1 paradigm from the La Heij et al. (23) study. The target stimuli were selected from Snodgrass and Vanderwart's (29) A Standardized Set of 260 Pictures (line pictures). Targets were selected in order of the following standards: ease of labeling, high level of familiarity, and consistency between targets and pictures. Consequently, 28 target stimuli were selected. To formulate abstract stimuli to use with the object stimuli, the complexity of the lines was divided into three levels and the abstract stimuli were created accordingly. The final object targets were colored red, yellow, blue, and green using Portable Photoshop 8 CS. In addition, seven self-injury tools were separately presented in Block 3 for further analysis. Among the line pictures of seven self-injury tools, three were selected from the previously mentioned papers and four were selected through an internet search.

After the participant sits down in front of the computer and wears the headset, verbal measurement sensitivity (delay & threshold) is tested using Voice Key. A fixation cross is then presented in a gray rectangular box (dimensions 14.37 cm × 8.54 cm) in the middle of a black screen, followed by random presentation of the object or abstract stimuli. The stimuli disappear when the first verbal syllable is recognized. When no speech is recognized, the stimuli are presented for up to 2,000 ms and disappear. The inter-stimulus interval is 500 ms, and the fixation cross is presented during the interval. Practice trials include 16 trials (8 object stimuli, 8 abstract stimuli), and experimental trials consist of two blocks, each block comprising 56 trials (28 object stimuli, 28 abstract stimuli). In the additional Block 3, the self-injury tools are presented as object stimuli. To reduce fatigue during experiment, there is a 10 s break between each block, and when the participant presses the space bar, the next block begins. When calculating for the mean reaction time per block, responses below 300 ms or above 1,500 ms were considered as errors (noise or omission error) and were excluded. In the practice trial stage, participants' sight problems (e.g., color-blindness) were identified, and correct/incorrect responses during performance were double checked using manual recording by the experimenter and screen and voice recording using the Open Broadcaster Software program.

### Data Analysis

Object-interference tasks were performed on 48 participants who reported repeated NSSI. In object-interference tasks, neurocognitive process affects speech production speed, producing difference under 200 ms. Therefore 3 cases showing outlier values larger than 2SDs were excluded for analysis, leaving a total of 25 people. Participants in the healthy control group were matched for age and sex with the NSSI group. Statistical analyses were performed using IBM SPSS Statistics 23. Descriptive analyses were performed on participants' demographic information and NSSI related characteristics. To identify for O-I effect between groups, mixed 2-way ANOVA of 2 between subjects (Groups) × 2 within subjects (Stimulus) was conducted. In addition, a 1 way-ANOVA was conducted to specifically examine the effects of stimulus type.

## Results

### Demographics and Clinical Characteristics

The prior homogeneity test confirmed homogeneity of gender and age by group in the sample (gender *X*^2^ = 0.303, *p* = 0.581; age *X*^2^ = 1.63, *p* = 0.204). The mean age of participants was 21.86 (*SD* = 2.62), and 82.2% were females. Based on the frequency of occurrence “within the last year” on the SITBI-K, 23 (51.5%) reported 5 or more NSSI experiences, satisfying satisfies diagnostic criterion A of NSSI in *Diagnostic and Statistical Manual of Mental Disorders (DSM)*-5. Based on 2 or more years as a standard for chronic NSSI, 33 (73.3%) reported chronic NSSI, which includes current remission as well as partial remission. This confirmed the appropriateness of the sample to test for O-I effect in repeated NSSI experiences.

### O-I Effect Between NSSI and Control Groups

**Figure 2:**
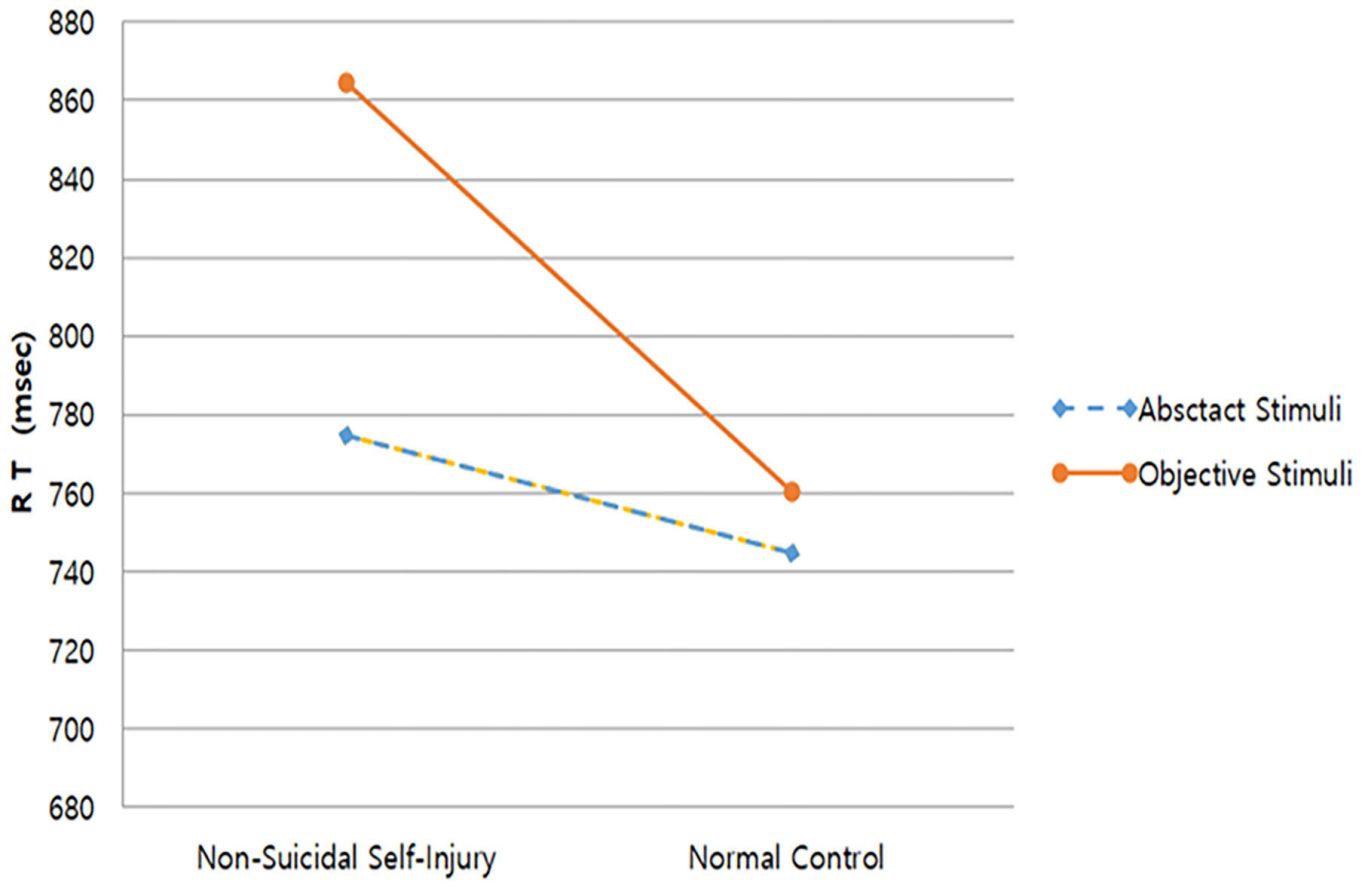
Reaction Times of Block 1 and 2.

The mean and standard deviations of O-I task performance for the NSSI and control groups are presented in Table 1. When analyses were performed on the basis of response times, significant main effect of stimulus condition was relatively greater [η^2^ = 0.38, *F*_(1, 88)_ = 33.34, *p* = 0.000], and the main effect of group [η^2^ = 0.09, *F*_(1, 88)_ = 7.90, *p* = 0.006] and interaction effect between group and stimulus condition [η^2^ = 0.09, *F*_(1, 88)_ = 10.22, *p* = 0.002] were also significant. Specifically, compared to reaction time difference between the object and abstract stimulus in the healthy control group [*F*_(1, 44)_ = 4.84, *p* = 0.033], the difference in the NSSI group was remarkably larger [*F*_(1, 44)_ = 30.61, *p* = 0.000]. Furthermore, reaction times were delayed for object stimulus compared to abstract stimulus (Figure 2). These results support the hypothesis, providing evidence of the O–I effect in repeated NSSI group.

**Table 1:** Results of object-interference task of NSSI and control group.

	**NSSI (*****n*** **=** **45)**				**NC (*****n*** **=** **45)**			
	**Object stimulus**		**Abstract stimulus**		**Object stimulus**		**Abstract stimulus**	
**Reaction**	***M***	**±*SD***	***M***	**±*SD***	***M***	**±*SD***	***M***	**±*SD***
Time (msec)	864.67	125.57	760.65	81.03	774.60	119.23	744.89	83.31
Error (n)	0.27	0.54	0.18	0.32	0.21	0.33	0.17	0.28

*NSSI, non-suicidal self-injury; NC, normal control; M, mean; SD, standard deviation*.

Analyses performed based on error rates revealed non-significant results for the main effects of group, stimulus types, as well as interaction between group and stimulus types. The NSSI group had a slightly greater mean number of errors but this was not statistically significant [*F*_(1, 88)_ = 0.291, *p* = 0.591].

### NSSI Tools Interference Task Effect on NSSI and Control Groups

In Block 3, NSSI tools were presented as object stimuli. The means and standard deviations of the NSSI tools interference task for the NSSI and control groups are presented in Table 2.

**Table 2:** Results of NSSI tools interference task in NSSI and control group.

	**NSSI (*****n*** **=** **45)**				**NC (*****n*** **=** **45)**			
	**Object stimulus (NSSI tools)**		**Abstract stimulus**		**Object stimulus (NSSI Tools)**		**Abstract stimulus**	
**Reaction**	***M***	**±*SD***	***M***	**±*SD***	***M***	**±*SD***	***M***	**±*SD***
Time (msec)	906.69	147.64	903.38	142.34	785.00	117.95	855.33	144.82
Error (n)	0.42	0.62	0.33	0.56	0.18	0.39	0.13	0.41

*NSSI, non-suicidal self-injury; NC, normal control; M, mean; SD, standard deviation*.

**Figure 3:**
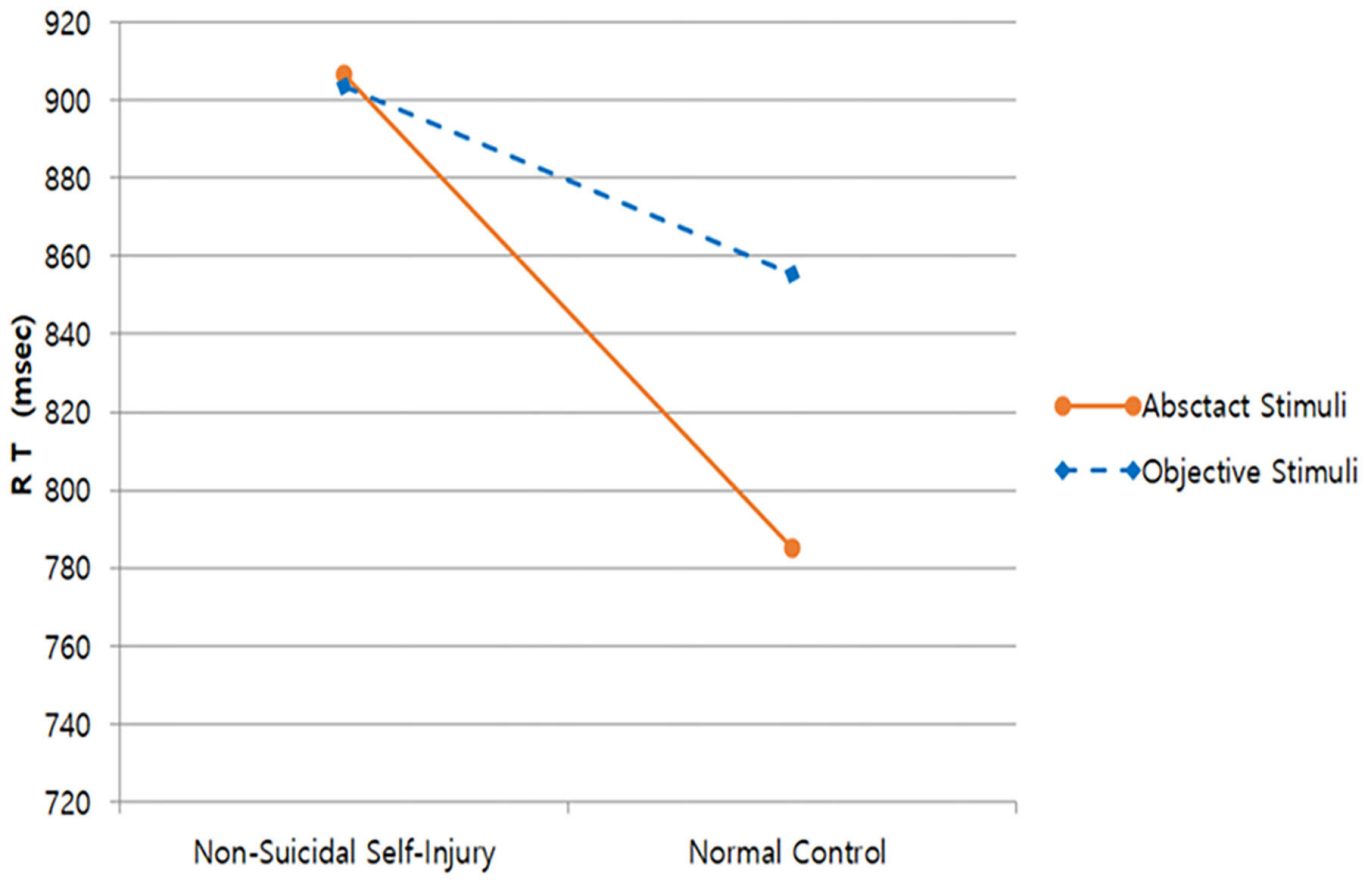
Reaction times of Block 3.

Based on the reaction times, the main effect of group [η^2^ = 0.14, *F*_(1, 88)_ = 12.33, *p* = 0.001], main effect of stimulus [η^2^ = 0.05, *F*_(1, 88)_ = 4.15, *p* = 0.045], and interaction effect between group and stimulus [η^2^ = 0.06, *F*_(1, 88)_ = 5.01, *p* = 0.028] were all significant. As for the NSSI group, the difference in reaction times between NSSI tool stimuli and abstract stimuli were not significant [*F*_(1, 44)_ = 0.02, *p* = 0.893]. On the other hand, reaction times were relatively longer for the abstract stimulus compared to NSSI tool stimuli in the control group [*F*_(1, 44)_ = 10.28, *p* = 0.003]. This is an unusual result, because the control group showed relatively healthier executive control abilities in Blocks 1 and 2 compared to the NSSI group. What is more noteworthy is that reaction times were delayed overall for the NSSI tools stimuli compared to reactions times in Blocks 1 and 2, for both groups (Figure 3).

**Figure 4:**
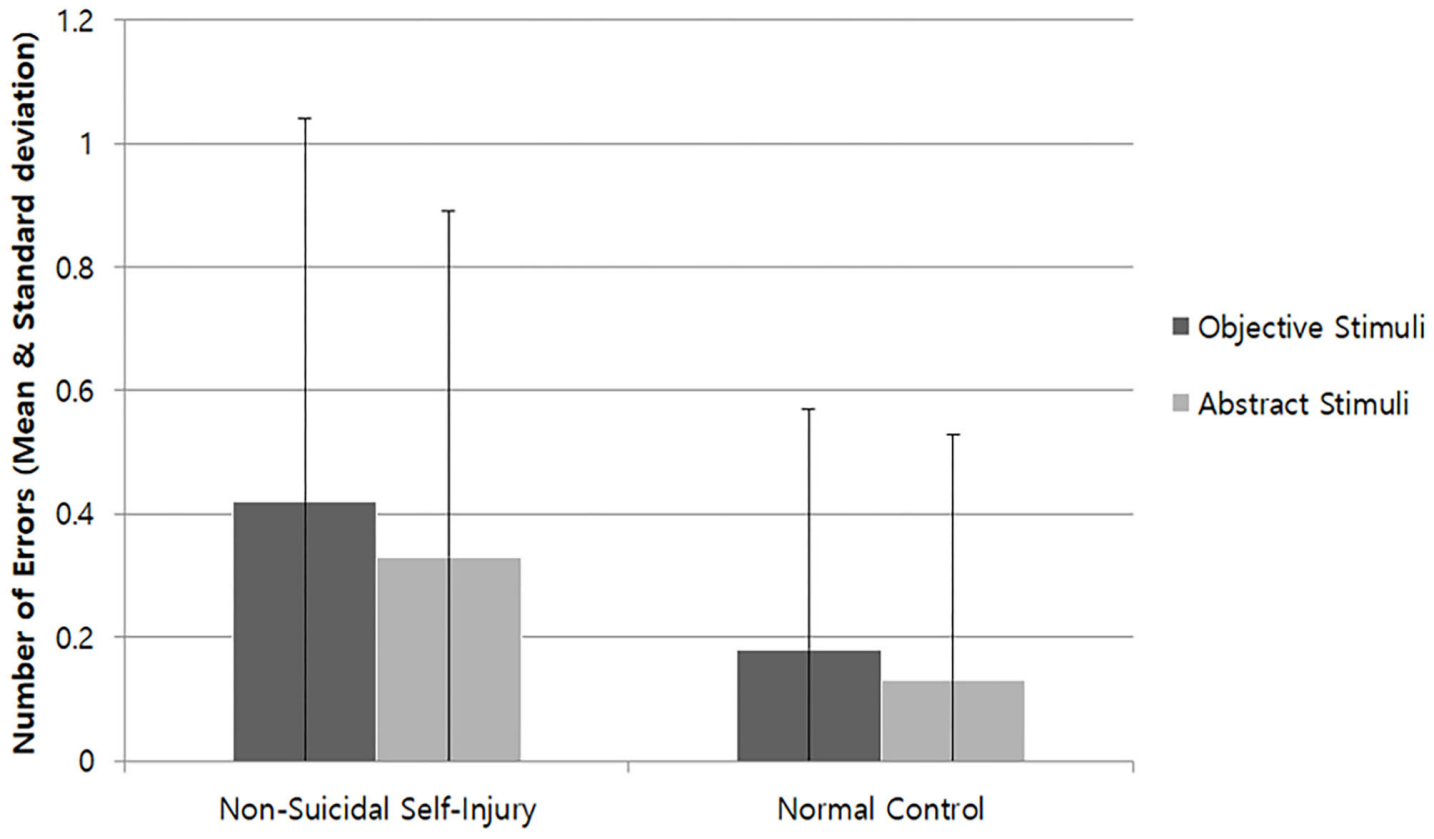
Number of Errors of Block 3.

In terms of the number of errors, the main effect of group was significant [η^2^ = 0.10, *F*_(1, 88)_ = 8.51, *p* = 0.004] but the main effect of stimulus conditions and the interaction effect between group and stimulus conditions were not. For the NSSI group, reaction times were significantly delayed without deviation for both the NSSI tool stimuli and abstract stimuli compared to the control group but had relatively higher errors (Figure 4).

## Discussion

This study attempted to investigate whether the O-I effect was present in those who have experienced repeated NSSI. Unlike the reaction times of the control group, the NSSI group displayed O-I effect characterized by longer reaction times for object stimuli compared to abstract stimuli. These results show a similar pattern with results found in previous research on children younger than 6.5 years with immature frontal lobe executive function development and OCD patients (19, 23). On the other hand, there was no significant difference for error rate. O-I effect affects neurocognitive processing, which manifests as a small difference of up to 200 ms in speech production speed. In other words, the O-I task is not difficult enough to show errors by participants with good neurocognitive abilities.

This study has the following clinical significance and treatment implications. Firstly, repeated NSSI experience is a type of habitual behavioral control problem, which can cause task confusion between object-induced behavior and goal-oriented behavior in everyday life. In the proposed NSSI diagnostic criteria for future study in the DSM-5 (30), diagnostic criterion A “In the last year, the individual has, on 5 or more days, engaged in intentional self-inflicted damage” is stated. This also leads to diagnostic criterion C “thinking about NSSI that occurs frequently, even when it is not acted upon” and diagnostic criterion E “the behavior or its consequences cause clinically significant distress or interference in interpersonal, academic, or other important areas of functioning.” The above criteria clearly relate to problems associated with repeated NSSI and not with NSSI as a one-off experience. The O-I effect in the NSSI group suggests deficits in task control abilities, as this group found it difficult to suppress the pressure induced from external objects found during daily goal-oriented behaviors, and experienced task confusion which hinders the original goal-oriented activities. This is in line with previous studies (7, 31), and thus, NSSI can be interpreted as a kind of behavioral addiction or compulsive behavior control problem.

Secondly, in the past DSM-IV-TR (32), OCD was classified as an anxiety disorder; trichotillomania (hair-pulling disorder) and excoriation disorder (skin-picking) as impulse control disorders; and NSSI behaviors as one of the symptoms for borderline personality disorder. However, during the transition to DSM-5 (30), disorders were re-classified based on accumulated findings, in consideration of clinical utility. OCD became independent from anxiety disorders to form obsessive-compulsive and related disorders, and trichotillomania and excoriation disorder were included here. This indicates grouping together of disorders characterized by “preoccupations and repetitive behaviors or mental acts in response to the preoccupations.” Furthermore, NSSI was newly added under “conditions for further study,” and it is mentioned that “when the behavior occurs frequently, it might be associated with a sense of urgency and craving, the resultant behavioral pattern resembling an addiction.” Therefore, with reference to this study's results, it is appropriate to suggest that NSSI will be incorporated into obsessive-compulsive and related disorders that are mainly characterized by preoccupations and pressure of repeated behaviors, or into non-material related disorders in substance-related or addiction disorders, characterized by repeated behaviors and cravings.

With regards to treatment, Klonsky and Glenn (11) has suggested that the most effective method to resist NSSI impulses is to remove the means of NSSI (tools) frequently used at home. This of course makes it physically impossible to self-injure but can also serve to prevent task confusion between automatic NSSI impulses triggered by related objects from surroundings and ongoing everyday tasks. Those who engage in repeated NSSI often report that merely keeping self-injury tools within reach is enough to feel comfort, as they are related to functional factors of self-injury (reinforcing factors). However, keeping self-injury tools close will increase the risk of self-injurious behaviors. Of course, it is impossible to remove all objects that can be used for self-injury from patients (in absence of frequently used tools, they may break objects such as plastic to create new tools), and external control by family, etc. is not a fundamental method to stop and change self-injurious behaviors (33). Although, in early stages of treatment where it is difficult to control urges to repeat self-injury, it is necessary to guide the patients to understand that removing self-injury tools is effective in preventing self-injurious behaviors.

Furthermore, recent studies consider the media, the Internet, peer group, etc., as priming factors for SITBs (21, 34), which trigger pain perception, attention bias, attitudes, and acting out related to NSSI. Therefore, it is imperative to prioritize removal of e means of NSSI (tools) in the treatment of NSSI, as well as deliberation regarding dealing with NSSI related information on the Internet and the media.

NSSI tools were also presented in Block 3 as object stimulus for additional analyses, which was expected to cause greater task confusion. As a result, while the difference between the object stimulus and abstract stimulus for NSSI group was not significant, reaction times for the abstract stimulus were significantly longer than for the object stimulus in the control group. There was also an unusual number of errors which was more than twice as high for the NSSI group compared to the control group. As mentioned previously, error number is not a sensitive measure for reaction times in individuals with good neurocognitive abilities, which was confirmed via non-significant error numbers for Blocks 1 and 2. Furthermore, it is notable that the overall reaction times in Block 3 were longer than Blocks 1 and 2. In this regard, considerations should be made to investigate how using NSSI tool stimulus in Block 3 may have affected the participants.

While objects such as a fruit knife, cutter knife, awl, scissors, etc., were presented as NSSI tool object stimuli, line pictures were used. This may have caused difficulties in perceiving the stimuli as threats or experiencing disgust on a conscious level. While it is difficult to predict the type of emotion induced from the object, it can be assumed that the stimuli may have had different emotional valence for the NSSI and control groups. In addition, since both participant groups were informed regarding the current study, it is possible that NSSI tools were perceived by similar attributes and functions, interfering with the performance due to ideological thinking.

As a result, both groups were hindered by neurocognitive performance, resulting in significantly longer reaction times. The unusual number of errors shown by the NSSI group reflects obvious cognitive mistakes, suggesting that the stimuli may have caused strong cognitive confusion in some way. Furthermore, the NSSI group showed significantly delayed reaction times for both NSSI tools and abstract stimuli, which may be due to strong cognitive confusion. For the control group, reaction times were unusually longer for abstract stimuli compared to NSSI stimuli, which is contrary to the O-I effect. This could be because there was a carry-over effect during a fast and simultaneous presentation of NSSI stimuli and abstract stimuli, producing mixed results. Of course, the NSSI tool stimuli in Block 3 is more of an exploratory investigation rather than based on sufficient theoretical background, therefore a more controlled research paradigm should be examined in the future, while supplementing for theoretical evidence.

The limitations of the current study are as follows. First, there is a sample limitation. The participants for this study were sampled through promoting the research in three universities in Seoul, therefore it is a somewhat limited representation of the entire population who experience NSSI. This study had the advantage of being able to achieve a homogeneous cognition level among participants as their neurocognitive abilities were measured. However, the participants are students from leading universities, who are presumably highly educated with high cognitive functioning limiting generalization. In addition, “those who reported serious NSSI that required medical treatment in the last month” were removed as per research ethics during sampling. This may limit generalization of the current results to those who experience serious levels of self-injury. Also, while most participants were experiencing repeated self-injury at the time, 12 participants were in full or partial remission (last NSSI within 4 years). Those currently repeating self-injuries and those who stopped self-injuries may create differences in their results. As it was difficult to compare their differences in the current study due to limited sample size, it needs to be considered in future studies.

Another limitation is that of the appropriateness of the NSSI tool stimulus used in Block 3. NSSI methods and tools that were used in the NSSI group were selected from previous studies (1, 11, 14), and this was not different from what the participants in the current study reported. For example, “cutting using a sharp object,” “burning” were reported as the highest rate, therefore a cutter knife, scissors, awl, lit cigarette, etc., were used as object stimulus. However, individuals who experience NSSI have different methods and tools for frequent NSSI. Therefore, the specific NSSI tools presented may hold a special significance for certain participants compared to a general object stimulus, but this may not be true for other participants, thus obscuring the results. The results of this study show that the response patterns of the two groups in Blocks 1 and 2 compared to Block 3 were quite different, leaving much room for discussion. If NSSI tools familiar to each individual were selectively presented, or selectively used during result analyses, they would have had a more accurate interpretive meaning, which should be supplemented in subsequent studies.

## Data Availability Statement

The raw data supporting the conclusions of this article will be made available by the authors, without undue reservation.

## Ethics Statement

The studies involving human participants were reviewed and approved by Chung-Ang University Institution Review Board (No. 1041078-201801-HRSB-019-01K). The patients/participants provided their written informed consent to participate in this study.

## Author Contributions

SL: conceptualization, methodology, validation, formal analysis, investigation, data curation, writing-original draft. MH: supervision, project administration, writing-review, and editing. Both authors contributed to the article and approved the submitted version.

## Conflict of Interest

The authors declare that the research was conducted in the absence of any commercial or financial relationships that could be construed as a potential conflict of interest.
